# Contributions of Physical Activity and Positive Psychological Functioning to Flow and Well-Being

**DOI:** 10.3390/sports13090301

**Published:** 2025-09-02

**Authors:** Nuno Rodrigues, Luís Sérgio Vieira, Cátia Sofia Martins, Catarina Moreira, Saúl Neves de Jesus

**Affiliations:** 1 University Research Center in Psychology (CUIP), Campus of Gambelas, University of Algarve, 8005-139 Faro, Portugal; lsvieira@ualg.pt (L.S.V.); snjesus@ualg.pt (S.N.d.J.); 2 Faculty of Human and Social Sciences, Campus of Gambelas, University of Algarve, 8005-139 Faro, Portugal; a71164@ualg.pt

**Keywords:** flow, physical activity, positive psychology, psychological well-being

## Abstract

Studies highlight the importance of physical activity (PA) in relation to positive psychological functioning (PPF) among adults. Physical inactivity is strongly associated with lower levels of PPF, supporting the idea that lifestyle choices can be identified as a public health concern. There is growing evidence of the health benefits of regular PA. This study aims to analyze the contribution of PA to flow, PPF, and well-being. The sample consisted of 226 adults aged between 18 and 65 years (*M* = 41.23; *SD* = 12.50), mostly female (70.35%), with 56% reporting regular PA. Results revealed significant differences favoring active individuals over sedentary participants in all dimensions of flow, except for loss of self-consciousness. Regular PA was associated with higher levels of flow and psychological well-being. Both regular and intensive PA, as well as environmental mastery (EM), were key contributors to flow experiences, while self-acceptance and EM were central contributors to the Live Well Index. These findings support the association between PA and lower likelihood of sedentary lifestyles, emphasize its benefits for well-being, and highlight the association between PPF and active lifestyle patterns.

## 1. Introduction

The World Health Organization [1,2,3] has declared sedentary lifestyles one of the greatest epidemics of the 21st century. Numerous scientific studies have concluded that physical inactivity entails substantial lifelong risks, while being physically active confers health benefits for individuals of all ages, genders, ethnic backgrounds, and socioeconomic statuses e.g., [1,4]. A systematic review aimed to identify the relation between the sedentary behavior (i.e., types) and several health-related outcomes found that sedentary behavior was negatively associated with cognitive functioning, physical activity levels, and health-related quality of life, and positively associated with depression and disability [5]. Research supports the idea that regular physical activity (PA) can be a decisive tool for improving physical fitness, quality of life, and ultimately, both physical and mental health [2,6].

Health is understood as a multidimensional human condition, encompassing physical, psychological, and social dimensions—each characterized by a continuum from negative to positive states. Positive health is associated with the ability to enjoy life and cope with challenges, beyond the mere absence of disease. In contrast, negative health is associated with illness and morbidity, potentially affecting various dimensions of functioning [7].

According to WHO [8], regular physical activity (PA) is essential for adults aged 18–64. It is recommended to engage in at least 150 min of moderate-intensity aerobic activity per week, or 75 min of vigorous-intensity activity, which can be accumulated in bouts of at least 10 min throughout the week. Additional health benefits can be achieved by increasing the duration or intensity of the activity. Moreover, adults should perform muscle-strengthening exercises involving major muscle groups two to three times per week.

Physically active individuals tend to enjoy higher levels of health, perform daily tasks with vigor and without excessive fatigue, and maintain sufficient energy to enjoy leisure activities and manage unforeseen demands. Moreover, they are at lower risk for several health problems and chronic diseases compared to inactive individuals [5,9,10].

Self-Determination Theory (SDT; [11,12]) has been widely applied to the PA context to explain human motivation, as it contributes to enjoyment and interest [12] and can play a central role in fostering intrinsic motivation [12,13]. When individuals engage in exercise of their own volition and interest, this reflects autonomous or intrinsic motivation [12]. Such activities are typically undertaken during leisure time and contrast with externally rewarded tasks (e.g., work). Intrinsic motivation is defined as “the doing of an activity for its inherent satisfactions rather than for some separable consequence. When intrinsically motivated, a person is moved to act for the fun or challenge entailed rather than because of external prods, pressures, or rewards” [11] (p. 56). It is associated with high-quality performance, creativity [12], and persistence [14]. People may engage in PA because they value its benefits for health (intrinsic reasons) or because it is socially recognized (extrinsic reasons) [12]. Thus, sports and PA are both sources of enjoyment and key contributors to health and well-being [5,12].

While intrinsic motivation is ideal for sustained engagement, individuals often participate in sport for multiple reasons. Intrinsic and extrinsic motives frequently coexist, shaping the overall quality of motivation and influencing behavioral outcomes [11,12]. This multidimensional approach to extrinsic motivation is based on internalization, where different regulation types fall along a continuum from external control to full integration with personal values. These types include external, introjected, identified, and integrated regulation, each reflecting increasing autonomy in motivation. These different motivations are reflected in distinct regulatory styles, which describe how individuals regulate their behavior along a continuum from autonomous to controlled motivation. Autonomous regulation includes intrinsic motivation and identified regulation, where actions are aligned with personal values and interests, while controlled regulation involves external and introjected regulation, driven by external demands or internal pressures such as guilt or obligation. In sports, more autonomous forms of motivation—such as intrinsic motivation and well-internalized extrinsic motivation—are linked to better persistence, performance, well-being, and adaptive behaviors [11,12,13]. Conversely, less autonomous motivation predicts negative outcomes like burnout, dropout, and negative self-talk [13].

Building on the understanding of motivation in physical activity, it is important to explore how these motivational processes relate to broader concepts of well-being and quality of life. The Live Well Index, developed by Marujo et al. [15] offers a broad and multifaceted framework for understanding quality of life, emphasizing a holistic perspective that goes beyond the absence of illness or material wealth. Designed for use in transnational studies, the index includes seven dimensions and is closely linked to factors such as motivation for physical activity. One of its key components, the general psychological well-being scale, captures individuals’ perceptions of their psychological state—an essential pillar of a well-lived life. This tool has been applied in studies across eight European countries, to explore how different motivational patterns influence engagement in PA. In particular, the Move Well dimension of the index highlights the role of physical activity in promoting well-being.

So, it is relevant to consider that people may engage in PA for intrinsic reasons (e.g., valuing its health benefits) or extrinsic reasons (e.g., social recognition). These motivations shape not only the frequency and intensity of participation but also the subjective experience of well-being derived from it. Thus, PA is not only a source of enjoyment but also a meaningful contributor to both psychological and physical health—an idea that aligns closely with the holistic vision of quality of life proposed by the Live Well Index.

Intrinsic motivation and autotelic experience—a central component of flow—are theoretically similar constructs. Autotelic activities are performed for their own sake, without expectation of external rewards [12,16]. Flow is described as an optimal psychological state characterized by intense focus and full engagement in an activity [17], and it appears frequently in the context of PA [18].

Flow can also be conceptualized through two complementary dimensions: situational flow, which arises from specific external conditions such as task structure or social context, and dispositional flow, which reflects a stable personality trait that predisposes individuals to experience flow across various settings [19,20,21]. This distinction is particularly relevant in the context of PA which inherently promotes flow experiences due to its structured nature, clear goals, and the balance between challenge and skill. These characteristics often lead to a sense of mastery and enjoyment [22].

Therefore, the nature of PA itself seems to promote flow experiences. This occurs because such activities often involve clear goals, constant skill-based challenges, and a sense of mastery and pleasure upon achievement [23]. Studies (e.g., [20,24,25]) have found positive associations between flow and PA, identifying determinants such as co-participant support [26], challenge-skill balance [18], situational motivation [27], physical self-concept [28], and body image [29]. Although the relationship between motivation (e.g., flow), regular PA, and psychological well-being is well established [30], it requires ongoing research, especially since lifestyle behaviors can change over time [31] and across the lifespan [32]. Previous research has shown that individuals engage in PA for different reasons, values, goals, and contextual circumstances [33], and these may vary across developmental stages [34].

Given this background, the present study aims to analyze the relationships among physical activity to flow, positive psychological functioning (PPF), and well-being. The specific objectives are as follows: (1) compare flow dimensions between physically active and sedentary individuals; (2) examine the relationships between PA, flow, and well-being; and (3) assess the predictive roles of PA and psychological well-being on flow. We also propose the following hypotheses:

**H1.**  *Individuals who engage in regular physical activity will report higher levels of flow experiences compared to those who are less physically active.*

**H2.**  *The autotelic experience will be positively associated with overall flow levels during physical activity.*

**H3.**  *Psychological well-being dimensions will significantly contributes to flow experiences among participants, beyond the effects of physical activity engagement.*

## 2. Methods

### 2.1. Participants

According to G*Power Version 3.1.9.3 [35] and to the recommendations regarding the normality assumptions, a sample between 34 and 120 participants was required [36]. A total of 226 adults, aged from 18 to 65 years (*M* = 41.23, *SD* = 12.50), mainly female (70.35%), participated. Participants were divided into two subgroups: 126 (55.75%) engaged in regular PA and 100 (44.25%) classified as non-active or sedentary (No PA). Half of the sample (*n* = 113; 50%) was drawn from the academic community of a regional University (including students, teaching staff, non-teaching staff, and others), while the remaining 113 participants (50%) came from outside the university and represented a variety of professional backgrounds. The types of regular PA reported by participants were diverse, ranging from individual sports (e.g., running, karate, swimming) and team sports (e.g., football, basketball, futsal) to group fitness classes (e.g., functional training, Zumba, Pilates, yoga) and water-based activities (e.g., sailing, surfing, bodyboarding).

For the purposes of this study, individuals were considered regular physical activity practitioners if they engaged in physical activity at least twice a week, for a minimum of 30 min per session, for more than six months.

### 2.2. Instruments

The practice of physical activity was characterized through a set of questions (e.g., “how long have you been practicing”; “on average, how many times do you practice per week and for how long”; “what activities do you practice”). The intensity was considered through a self-report instrument, based on Borgs’ measure [37,38] with the classification between 1 (i.e., low intensity) and 10 (i.e., high intensity); scores between 1 and 5 were considered as low/moderate intensity and from 6 to 10 as moderate/high intensity.

To assess the various psychological dimensions, we used the following scales: the Ryff Scale of Psychological Well-Being, the Dispositional Flow Scale 2 and the Live Well Index. In their adaptation for the Portuguese population, all of them underwent content validity through Confirmatory Factor Analysis [15,39,40].

#### 2.2.1. Ryff Scale of Psychological Well-Being

To assess psychological well-being, a 42-items of the Ryff Scale of Psychological Well-Being (SPWB; [34,39]) was used, which describes the multidimensional construct of positive psychological functioning in the six dimensions described by the author: autonomy (7 items; e.g., “I am not afraid to voice my opinions, even when they are in opposition to the opinions of most people”; α = 0.69), environmental mastery (7 items; e.g., “In general, I feel I am in charge of the situation in which I live”; α = 0.67), personal growth (7 items; e.g., “I have a sense that I have developed a lot as a person over time”; α = 0.62), positive relations with others (7 items; e.g., “Most people see me as loving and affectionate”; α = 0.71), self-acceptance (7 items; e.g., “In general, I feel confident and positive about myself”; α = 0.78), and purpose in life (7 items; e.g., “I enjoy making plans for the future and working to make them a reality”; α = 0.64). The answers were given according to a 6-point Likert-type scale (1—Completely Disagree to 6—Completely Agree). Higher scores reveal higher levels of the respective dimension.

#### 2.2.2. Dispositional Flow Scale—2

The flow construct was measured using a Portuguese version [40] of the Dispositional Flow Scale—2 (DFS-2; [20]) composed of 36 items that assessed the nine dimensions of the construct: (1) challenge-skill balance (4-items; e.g., “I am challenged, but I believe my skills will allow me to meet the challenge”; α = 0.81); (2) action-awareness merging (4-items; e.g., “I make the correct movements without thinking about trying to do so”; α = 0.81); (3) clear goals (4-items; e.g., “I know clearly what I want to do”; α = 0.83); (4) unambiguous feedback (4-items; e.g., “It is really clear to me how my performance is going”; α = 0.85); (5) concentration on the task (4-items; e.g., “My attention is focused entirely on what I am doing”; α = 0.72); (6) sense of control (4-items; e.g., “I have a sense of control over what I am doing”; α = 0.83); (7) loss of self-awareness (4-items; e.g., “I am not concerned with what others may think of me”; α = 0.91); (8) time transformation (4-items; e.g., “The way time passes seems to be different from normal”; α = 0.80); and (9) autotelic experience (4-items; e.g., “I really enjoy the experience”; α = 0.91). Answers were given using a 5-point Likert scale (1—Never to 5—Always). Higher scores reveal higher levels of the respective dimension.

#### 2.2.3. Live Well Index

It is a 10-item instrument (Live Well Index; [15]) where each item corresponded to a statement related to the psychological dimension within the scope of a life with well-being (e.g., “I am satisfied with my life”; α = 0.93). The answers were given through a 10-point Likert-type scale, from 1—I totally disagree to 10—I totally agree. Higher scores reveal higher levels of the respective dimension.

### 2.3. Procedures

Ethical approval was obtained from the Department of Psychology and Educational Sciences at the University of Algarve (Reference No. E-UAlg/2017/20222). In addition, formal consent was obtained from the original authors of the instruments used.

Participants in the regular physical activity (PA) group were recruited from individuals enrolled in group classes organized by the University Sports Office, as well as from other settings offering PA opportunities (e.g., gyms, associations, and sports clubs) in the Algarve region. Other participants were contacted through several services of the university (e.g., management, social services, library) and the general community.

Data collection was carried out through an aggregated questionnaire composed of the instruments previously described. Upon approaching participants, the purpose and objectives of the study were explained, and informed consent was obtained. Participants were assured of the confidentiality and anonymity of their responses.

A regular PA practitioner was defined as an individual who engages in physical activity at least twice a week, for a minimum of 30 min per session, over a period of more than six months [1,2,3]. To ensure that responses on the flow measure referred specifically to PA, participants were asked to answer in relation to the physical activity they practiced most frequently at the time (in the case of regular PA practitioners) or had practiced in the past (in the case of non-regular PA practitioners).

### 2.4. Statistical Analysis

Data were analyzed using the IBM SPSS version 30.0.1. [41], and tests of normality were conducted. Since most variables demonstrated normality, the remaining variables were examined for kurtosis and skewness. As their values did not exceed critical thresholds [36], parametric statistics were used. Internal consistency was assessed through Cronbach’s alpha, with values above 0.60 considered adequate and above 0.90 as excellent [36,42]. Descriptive statistics were computed, and independent *t*-tests were used to test the differences between the two groups with different physical practices, with a significance level of 0.05. Hedges’ *g* was computed to assess effect size, with values interpreted as small (*g* = 0.20–0.49), medium (*g* = 0.50–0.79), and large (*g* ≥ 0.80). Correlations were also used to evaluate the associations between the continuous variables, and multiple regression analysis were conducted by blocks using the Stepwise method to assess the relation between variables. Regarding multiple regression assumptions, we considered that: (i) the number of participants corresponds to more than 20 to 25 participants per independent variable selected; (ii) the correlation among independent variables were below 0.70; (iii) collinearity statistics and tolerance values ranged between 0.797 and 0.963, for each independent variable across all regression models, suggesting no multicollinearity issues. A significance level of 0.05 was considered [35]. Block 1 included age, block 2 included regular physical activity (1—no; 2—yes) and intensity of physical activity, and block 3 included all dimensions of positive psychological functioning. Only significative blocks were reported.

Given the exploratory nature of this study, stepwise regression was employed to identify potential correlations between flow and psychological well-being. We acknowledge that this method can capitalize on sample-specific variance and increase the risk of overfitting, especially with a relatively modest sample size and some variables showing marginal reliability. Therefore, findings should be interpreted with caution, and these analyses serve primarily as hypothesis-generating rather than confirmatory.

Finally, it is important to note that all variables were measured simultaneously through a cross-sectional survey. Thus, while regression analyses were used to examine relationships among variables, no temporal or causal inferences can be made.

## 3. Results

As Table 1 shows, the results reveal average levels of flow dimensions and total scores, where time transformation (*M* = 3.65; *SD* = 0.76) present the lower mean and the autotelic experience (*M* = 4.30; *SD* = 0.69) the highest one. Regarding the range, the minimum levels vary between 1.00 (loss of self-consciousness) and 2.42 (total flow), and the maximum is 5.00 for all assessed domains.

The comparison between non-regular PA practitioners (sedentary individuals) and regular PA practitioners (Table 2) revealed significant differences across all dimensions except for loss of self-consciousness (*t* = 0.60, *p* = 0.953, *g* = 0.01). The effect sizes ranged from small to large (*g* = 0.31–0.76). Overall, the results indicate that participants who engage in regular physical activity exhibit higher levels across the measured dimensions compared to those who do not.

The relationships between flow and its dimensions, the various psychological well-being dimensions, the general Live Well indicator, age, and physical activity (PA) intensity were analyzed (Table 3). Participants’ age showed significant negative associations with PA intensity (*r* = −0.37, *p* ≤ 0.01), personal growth (*r* = −0.28, *p* ≤ 0.01), and autotelic experience (*r* = −0.28, *p* ≤ 0.05), as well as a significant positive association with environment mastery (*r* = 0.25, *p* ≤ 0.01). These findings indicate that older individuals tend to report lower levels of PA intensity, personal development, and autotelic disposition in PA, but higher perceived control over their environment.

The intensity of physical activity showed small but significant positive correlations with most of the assessed dimensions, ranging from *r* = 0.16 (*p* ≤ 0.05, action-awareness merging) to *r* = 0.39 (*p* ≤ 0.01, challenge-skill balance). No significant correlations were found between PA intensity and the flow dimensions of concentration, loss of self-awareness, and time transformation.

All dimensions of psychological well-being were positively and significantly correlated with each other and with the total flow score, with correlations ranging from *r* = 0.20, (*p* ≤ 0.05) to *r* = 0.33 (*p* ≤ 0.01). Additionally, psychological well-being dimensions were positively associated with specific flow components, including action-awareness merging (*r* = 0.18 to 0.29, *p* ≤ 0.05–0.01), clear goals (*r* = 0.36 to 0.48, *p* ≤ 0.01), challenge-skill balance (*r* = 0.28 to 0.33, *p* ≤ 0.01), concentration (*r* = 0.21 to 0.34, *p* ≤ 0.05–0.01), sense of control (*r* = 0.26 to 0.36, *p* ≤ 0.01), unambiguous feedback (*r* = 0.31 to 0.46, *p* ≤ 0.01), and autotelic experience (*r* = 0.31 to 0.42, *p* ≤ 0.01).

The flow dimension of time transformation showed significant positive correlations only with positive relations (*r* = 0.24, *p* ≤ 0.01), environment mastery (*r* = 0.27, *p* ≤ 0.01), and autonomy (*r* = 0.25, *p* ≤ 0.01). Conversely, the loss of self-awareness dimension showed significant negative correlations with purpose in life (*r* = −0.20, *p* ≤ 0.05) and personal growth (*r* = −0.20, *p* ≤ 0.05).

Finally, the Live-well total score revealed moderate to high positive significant correlations with all dimensions of psychological well-being (ranging from *r* = 0.43, *p* ≤ 0.01, and *r* = 0.74, *p* ≤ 0.01), and with most flow dimensions (ranging from *r* = 0.22, *p* ≤ 0.05 to *r* = 0.43, *p* ≤ 0.01), except for positive relationship, personal growth and autonomy (Table 3).

To assess the contributive power of psychological well-being on flow among regular physical activity (RPA) practitioners, a multiple linear regression was conducted. Based on the correlation matrix, all dependent variables associated with the flow dimensions, (except loss-self-consciousness), the total flow and the live-well score were included. As shown in Table 4 and Table 5, all tested models were statistically significant.

Regarding the positive psychological functioning, participants’ age showed a significant association only with the challenge-skill balance (CSB) model (*β*_B2_ = 0.20; *β*_B3_ = 0.27, *p* ≤ 0.001). In this model (Table 4), intensity of PA (IPA) was significantly associated with the outcome across all blocks (*β*_B1_ = 0.40; *β*_B2_ = 0.36; *β*_B_ = 0.30, *p* ≤ 0.001), and environment mastery emerged as the only psychological variable significantly linked to positive psychological functioning (*β*_B3_ = 0.27, *p* ≤ 0.001). This model explained 26% of the variance (*p* ≤ 0.001), making it the most explanatory among the tested models.

The clear goals (CG) model is the one of the second most explanatory (*R*^2^ = 0.22, *p* ≤ 0.001), with IPA (*β*_B2_ = 0.30; *β*_B_ = 0.22, *p* ≤ 0.001), environment mastery (*β*_B_ = 0.24, *p* ≤ 0.001) and personal growth (*β*_B_ = 0.20, *p* ≤ 0.001) showing significant contributions. Similarly, the autotelic experience (AE) model also explained 22% of the variance (*R*^2^ = 0.22, *p* ≤ 0.001), with two relevant contributing variables: IPA (*β*_B1_ = 0.40; *p* ≤ 0.001; *β*_B2_ = 0.35; *p* ≤ 0.001) and personal growth (PG; (*β*_B2_ = 0.26; *p* ≤ 0.001).

The third most explanatory model was unambiguous feedback (UF; *R*^2^ = 0.19, *p* ≤ 0.001), which included three variables significantly associated with the outcome: IPA (*β*_B1_ = 0.20; *p* ≤ 0.001; *β*_B2_ = 0.22; *p* ≤ 0.001; *β*_B3_ = 0.23; *p* ≤ 0.001), EM (*β*_B2_ = 0.23; *p* ≤ 0.001; *β*_B3_ = 0.30; *p* ≤ 0.001), and self-acceptance (SA; *β*_B3_ = 0.17; *p* ≤ 0.05).

The overall flow model showed significant contributions from ME (*β*_B1_ = 0.33; *p* ≤ 0.001; *β*_B2_ = 0.29; *p* ≤ 0.001; *β*_B3_ = 0.21; *p* ≤ 0.001), IPA (*β*_B2_ = 0.26; *p* ≤ 0.001; *β*_B3_ = 0.26; *p* ≤ 0.001), and positive relationships (PR; *β*_B3_ = 0.17; *p* ≤ 0.05), explaining 20% of variance (*p* ≤ 0.001) of flow.

Finally, the live-well model revealed three main contributors: SA (*β*_B1_ = 0.66; *p* ≤ 0.001; *β*_B2_ = 0.48; *p* ≤ 0.001; *β*_B3_ = 0.42; *p* ≤ 0.001), EM (*β*_B2_ = 0.31; *p* ≤ 0.001; *β*_B3_ = 0.26; *p* ≤ 0.001), and purpose of life (PL; *β*_B3_ = 0.17; *p* ≤ 0.05), together explaining 52% of the variance (*p* ≤ 0.001).

Overall, in terms of significant contributors (Table 4 and Table 5), IPA (*β_B1_CCB & B1_AE_* = 0.40, *p* ≤ 0.001) and EM (*β_B1_Flow Tt & B2_CG_* = 0.33, *p* ≤ 0.001) emerged as consistently relevant variables. Within the live well model, SA was the most relevant contributor (*β*_Range_ = 0.42–0.66).

## 4. Discussion

Given this background, the present study aimed to explore the contribution of physical activity (PA) to flow, positive psychological functioning (PPF), and well-being. Specifically, the objectives were to (1) compare flow dimensions between physically active and sedentary individuals; (2) examine the relationships among PA, flow, and well-being; and (3) assess the contributing roles of PA and psychological well-being on flow.

Regarding the first objective, and in line with Hypothesis 1, findings revealed that individuals who regularly engage in physical activity reported significatively higher levels of flow across all dimensions, except for loss self-consciousness. These results are in line with previous studies that position PA—particularly when performed regularly and with moderate to vigorous intensity—as a privileged context for flow experiences [25,43,44]. This supports the view that PA provides the optimal balance between challenge and skill, as well as conditions such as clear goals and immediate feedback, which are essential for flow emergence [45,46]. The association between PA and flow is especially relevant given the potential of flow to reinforce the enjoyment and sustainability of active lifestyles, thereby counteracting the well-documented risk of exercise dropout [47,48].

In relation to the second objective, our results revealed significant and positive associations between PA, flow dimensions, and psychological well-being indicators—specifically in dimensions such as environmental mastery, positive relationships, autonomy, and personal growth. This pattern supports Hypothesis 2, which proposed that autotelic experience—a core component of flow—would be positively associated with overall flow levels during PA. The finding aligns with recent evidence suggesting that flow not only contributes to psychological well-being but is also facilitated by it (e.g., [4,49,50,51]). More specifically, individuals with greater psychological resources—such as a sense of purpose, autonomy, and emotional stability—may be more likely to experience immersion and intrinsic enjoyment in goal-directed activities, including physical activity. The reciprocal nature of this relationship highlights the dynamic interplay between individual well-being and optimal functioning in everyday experiences [52].

Finally, addressing the third objective, the regression models confirmed Hypothesis 3, showing that psychological well-being dimensions—especially environmental mastery, self-acceptance, and personal growth—significantly contribute to flow dimensions, such as autotelic experience, clear goals, and challenge-skill balance. These dimensions are considered core markers of intrinsically motivated flow experiences [46], and their association with PPF suggests that well-being acts as both a condition and an outcome of optimal experience. In turn, PA intensity emerged as a consistent contributor of flow, confirming that the physiological engagement and cognitive focus inherent to more vigorous physical activity may be particularly conducive to flow states [53]. In addition, sociodemographic variables, the intensity of physical activity, and regular engagement in physical activity significantly contributed to the explanation of several flow dimensions. These findings are consistent with the perspective of Gouveia [23,40] who argues that the very nature of physical and sport activities—characterized by goal-directedness, skill-based challenges, and immediate feedback—creates optimal conditions for flow to emerge. Particularly, higher intensity levels often provide more cognitively and physically demanding contexts, which stimulate focused attention, foster the setting of clear objectives, and enhance the intrinsic satisfaction derived from task accomplishment.

These findings support recent theoretical perspectives that position flow as a bridge between behavioral engagement and eudaimonic well-being [54,55]. Flow appears not only to facilitate pleasure and task involvement, but also to contribute to long-term flourishing through its links with autonomy, mastery, and purpose in life. In this sense, the Live-well total score—which integrates multiple components of well-being—was significantly correlated with both behavioral (PA) and psychological variables, reinforcing the integrative value of flow as an indicator of holistic functioning.

This study contributes to the growing literature emphasizing the central role of flow in promoting active and meaningful lifestyles. By showing that both physical activity and psychological well-being jointly contribute to optimal experience and functioning, our results highlight the importance of multidimensional approaches in interventions aimed at enhancing health, motivation, and personal development through physical activity.

A key and novel contribution of this study lies in the identification of specific contributors to flow, particularly the strong explanatory power of certain dimensions of psychological well-being (especially environmental mastery), along with regular participation in physical activity and its intensity. These results highlight the dual role of psychological well-being and the intrinsic features of physical activity in promoting active lifestyles and sustaining them over time. This suggests the need to incorporate motivational variables into the design of physical activity interventions. When individuals feel intrinsically motivated and perceive themselves as competent, they are more likely to experience flow. In turn, frequent flow experiences may be linked with higher intrinsic motivation and continued engagement in physical activity, thereby reducing dropout rates—which remain a major challenge in health promotion programs.

These findings suggest practical recommendations for intervention design. Programs aiming to increase physical activity participation could benefit from incorporating psychological skills training that enhances autonomy, goal setting, and self-acceptance. Ensuring that activities provide clear goals and immediate feedback may also help participants achieve flow states more consistently, thereby increasing adherence and long-term well-being.

Beyond aligning with existing literature, these results may also reflect how structured, goal-oriented physical activity contexts help individuals develop psychological resources such as autonomy and environmental mastery. This suggests that interventions should not only promote physical engagement but also explicitly support psychological needs to maximize well-being benefits. Additionally, the strong associations with environmental mastery indicate that a sense of control over one’s environment may be a critical leverage point for designing effective health promotion strategies.

Regarding limitations, this study reveals several concerns that will be addressed. First, the sample was not stratified by socioeconomic status, occupational background, or education level, which limits the generalizability of the findings. Future studies should ensure more representative sampling or conduct subgroup analyses to understand how these factors may influence the experience of flow and well-being in physical activity contexts.

Another limitation refers to the low internal consistency observed in certain subscales, which may have introduced measurement error, potentially attenuating the strength of associations detected in the regression analyses. Although some subscales, such as Personal Growth, presented Cronbach’s alpha values slightly below the conventional threshold (e.g., α = 0.62), they were retained due to their strong theoretical relevance and prior validation in the literature. Given the exploratory nature of this study, the inclusion of subscales with marginal reliability was considered acceptable, provided this limitation is acknowledged transparently. Future research should aim to use refined instruments with stronger psychometric properties to ensure more reliable modeling of these relationships.

A further limitation concerns the absence of detailed regression diagnostics, including formal tests for residual normality and homoscedasticity. While multicollinearity was addressed, failing to examine these additional assumptions may limit the interpretability and validity of the regression results. Future research should incorporate such diagnostics to strengthen methodological rigor. Also, the use of stepwise regression, which can inflate Type I error rates and reduce the external validity of findings by capitalizing on sample-specific variance. While this approach was selected to explore potential contributors in an under-researched area, future research should prioritize theoretically driven models and more conservative variable selection methods to improve generalizability.

Future research should consider longitudinal designs to further validate the associations and causal pathways identified here, particularly regarding the role of flow and psychological well-being in promoting adherence and maintenance of more active and autonomous behaviors.

## 5. Conclusions

Studying flow and other optimal experiences in the context of physical activity and sports practice is essential not only to understand the positive affective responses associated with these practices but also to identify the psychological processes that underlie adherence, persistence, and the long-term adoption of active and healthy lifestyles.

In this study, results showed that dispositional flow is positively associated with regular physical activity in nearly all dimensions. Regular practitioners exhibited significantly higher levels of flow than sedentary individuals in eight out of the nine flow dimensions, apart from loss of self-consciousness. Considering that flow is a key mechanism for sustaining behavioral engagement in physical activity and sport, this finding suggests the potential value of including flow-promoting elements into physical activity programs to foster long-term commitment and lifestyle integration.

## Figures and Tables

**Table 1:** Descriptive analysis of the dimensions and total scores of participants’ flow (*N* = 226).

Dimensions	*M*	*SD*	*Min*	*Max*
Challenge-skill balance	3.70	0.63	2.00	5.00
Action-awareness merging	3.65	0.63	1.75	5.00
Clear goals	4.09	0.64	2.25	5.00
Concentration	3.76	0.64	2.00	5.00
Sense of control	3.78	0.58	2.25	5.00
Unambiguous feedback	3.80	0.62	2.00	5.00
Loss self-consciousness	3.77	1.03	1.00	5.00
Time transformation	3.65	0.76	1.50	5.00
Autotelic experience	4.30	0.69	1.75	5.00
Total flow	3.83	0.49	2.42	5.00

Note. *M* = Mean, *SD* = Standard deviation, *Min* = Minimum, *Max* = Maximum.

**Table 2:** Comparison of the levels of flow between groups with regular practice of physical activity (PA) and no regular practice (No PA).

Dimensions	No PA (*n* = 100)		PA (*n* = 126)		*t*	*p*	*g*
	*M*	*SD*	*M*	*SD*			
Challenge-skill Balance	3.47	0.61	3.89	0.58	−5.23	<0.001	0.69
Action-awareness	3.54	0.64	3.74	0.60	−2.42	0.016	0.31
Clear goals	3.93	0.68	4.22	0.57	−3.53	0.001	0.46
Concentration	3.65	0.65	3.85	0.61	−2.42	0.016	0.32
Sense of control	3.67	0.63	3.87	0.53	−2.64	0.009	0.35
Unambiguous feedback	3.62	0.65	3.95	0.55	−4.05	<0.001	0.53
Loss self-consciousness	3.76	1.01	3.78	1.06	0.060	0.953	0.01
Time transformation	3.50	0.79	3.77	0.72	−2.74	0.007	0.36
Autotelic experience	4.02	0.71	4.52	0.59	−5.71	<0.001	0.76
Total flow	3.68	0.51	3.95	0.44	−4.21	<0.001	0.56

Notes. No PA = Without Physical Activity, PA = With Physical Activity, *M* = Mean, *SD* = Standard deviation, *t* = statistic test, *p* = significance level, *g* = effect size.

**Table 3:** Correlations between sociodemographic, physical activity, psychological well-being and flow (*N* = 226).

	2	3	4	5	6	7	8	9	10	11	12	13	14	15	16	17	18	19
1—Age	−0.37 **	−0.04	0.04	0.06	0.25 **	−0.24 **	0.07	0.15	−0.03	−0.09	−0.28 **	−0.01	−0.06	0.00	0.08	0.02	−0.21 *	−0.11
2—Int PA		0.22 *	0.20 *	0.19 *	0.20 *	0.38 **	0.24 **	0.20 *	0.16 *	0.27 **	0.39 **	0.14	0.30 **	0.27 **	−0.07	0.13	0.36 **	0.31 **
3—PR			0.46 **	0.52 **	0.55 **	0.49 **	0.46 **	0.45 **	0.26 **	0.38 **	0.33 **	0.26 **	0.30 **	0.39 **	−0.05	0.24 **	0.32 **	0.31 **
4—PL				0.50 **	0.52 **	0.53 **	0.41 **	0.57 **	0.18 *	0.48 **	0.28 **	0.26 **	0.30 **	0.37 **	−0.20 *	0.06	0.40 **	0.20 *
5—RPA					0.64 **	0.45 **	0.59 **	0.74 **	0.21 *	0.39 **	0.29 **	0.29 **	0.32 **	0.41 **	0.02	0.11	0.32 **	0.30 **
6—EM						0.45 **	0.55 **	0.62 **	0.29 **	0.44 **	0.31 **	0.34 **	0.36 **	0.46 **	0.05	0.27 **	0.31 **	0.33 **
7—PG							0.43 **	0.43 **	0.20 *	0.46 **	0.37 **	0.21 *	0.26 **	0.31 **	−0.20 *	0.12	0.42 **	0.28 **
8—AU								0.58 **	0.24 **	0.36 **	0.33 **	0.33 **	0.32 **	0.36 **	−0.00	0.25 **	0.32 **	0.27 **
9—LWT									0.16	0.43 **	0.22 *	0.22 *	0.31 **	0.43 **	−0.06	0.07	0.33 **	0.41 **
10—AAM										0.56 **	0.60 **	0.53 **	0.61 **	0.67 **	0.30 **	0.36 **	0.37 **	0.77 **
11—CG											0.63 **	0.56 **	0.64 **	0.73 **	0.19	0.29 **	0.59 **	0.80 **
12—CSB												0.57 **	0.57 **	0.62 **	0.04	0.44 **	0.57 **	0.76 **
13—C													0.60 **	0.58 **	0.23 **	36 **	0.48 **	0.79 **
14—SC														0.72 **	0.22 *	0.35 **	0.50 **	0.83 **
15—UF															0.11	0.39 **	0.52 **	0.84 **
16—LSC																0.21 *	−0.14	0.45 **
17—TT																	0.31 **	0.62 **
18—AE																		0.69 **
19—*Flow*																		-

Notes. * *p* ≤ 0.05; ** *p* ≤ 0.01; Int PA—Intensity of Physical Activity; PR—Positive relations; PL—Purpose in life; RPA—Regular Physical Activity Practitioner (1—no; 2—yes); EM—Environment mastery; PG—Personal Growth; AU—Autonomy; LWT—Life Well total score; AAM—Action-awareness merging; CG—Clear goals; CSB—Challenge-skill balance; C—Concentration; SC—Sense of control; UF—Unambiguous feedback; LSC—Loss of self-consciousness; TT—Time transformation; AE—Autotelic experience.

**Table 4:** Hierarchical regression for the dependent variables associated with the different dimensions of flow and live well index regarding considering PA.

VD	CSB			AAM		CG			C			SC			UF		
Model (VIs)	1	2	3	1	2	1	2	3	1	2	3	1	2	3	1	2	3
Age		0.20 **	0.27 **														
RPA				17 **	14 *												
IPA	0.40 **	0.36 **	0.30 **			0.29 **	0.30 **	0.22 **	0.17 **	0.13 *	0.12	0.22 **	0.18 **	0.17 **	0.20 **	22 **	0.23 **
PR											0.16 *			0.15 *			
PL																	
SA																	0.17 *
EM			0.27 **		0.24 **		0.33 **	0.24 **		0.30 **	0.22 **		0.25 **	18 *		0.23 **	0.20 **
PG								0.20 **									
AU																	
*R* ^2^	0.16	0.19	0.26	0.03	0.08	0.09	0.19	0.22	0.030	0.12	0.13	0.05	0.11	0.13	0.09	0.17	0.19
*R*^2^ Aj	0.15	0.19	0.25	0.03	0.08	0.09	0.18	0.21	0.025	0.11	0.12	0.04	0.10	0.11	0.08	0.17	0.18
*R*^2^ Δ	0.16	0.04	0.07	0.03	0.05	0.09	0.11	0.029	0.030	0.09	0.02	0.05	0.06	0.02	0.09	0.09	0.02
*Z*	41.04	26.66	26.06	6.69	10.10	21.06	26.18	20.73	6.77	14.36	11.22	10.85	13.41	10.44	21.22	23.12	17.43
*p*	0.000	0.000	0.000	0.010	0.000	0.000	0.000	0.000	0.010	0.000	0.000	0.001	0.000	0.000	0.000	0.000	000

Notes. * *p* ≤ 0.05; ** *p* ≤ 0.01; RPA—Regular Physical Activity (1—no; 2—yes); IPA—Intensity of Physical Activity; PR—Positive Relationships; PL—Purpose in Life; SA—Self-acceptance; EM—Environment mastery; PG—Personal Growth; AU—Autonomy; *R*^2^ Aj—Adjusted *R*^2^; *R*^2^ ∆—*R*^2^ Change.

**Table 5:** Hierarchical regression for the dependent variables associated with the different dimensions of flow and live well index regarding considering PA.

VD	TT		AE		Flow Total			Live Well		
Model VI	1	2	1	2	1	2	3	1	2	3
Age										
RPA	0.18 **	0.17 **								
IPA			0.40 **	0.35 **		0.26 **	0.26 **			
PR		0.20 **					0.17 *			
PL										0.17 *
SA								0.66 **	0.48 **	0.42 **
EM					0.33 **	0.29 **	21 **		0.31 **	0.26 **
PG				0.26 **						
AU										
*R* ^2^	0.03	0.07	0.16	0.22	0.11	0.18	0.20	0.44	0.50	0.52
*R*^2^ Aj	0.03	0.06	0.16	0.22	0.11	0.17	0.19	0.44	0.50	0.51
*R*^2^ Δ	0.03	0.04	0.16	0.06	0.11	0.07	0.02	0.44	0.06	0.02
*Z*	7.24	8.43	42.03	31.83	27.45	23.99	18.36	170.82	108.64	77.50
*p*	0.008	0.000	0.000	0.000	0.000	0.000	0.000	0.000	0.000	0.000

Notes. * *p* ≤ 0.05; ** *p* ≤ 0.01; RPA—Regular Physical Activity (1—no; 2—yes); IPA—Intensity of Physical Activity; PR—Positive Relationships; PL—Purpose in Life; SA—Self-acceptance; EM—Environment mastery; PG—Personal Growth; AU—Autonomy; *R*^2^ Aj—Adjusted *R*^2^; *R*^2^ ∆—*R*^2^ Change.

## Data Availability

The data can be made available for consultation upon request to the corresponding author.
